# Molecular Structure of Paraoxonase-1 and Its Modifications in Relation to Enzyme Activity and Biological Functions—A Comprehensive Review

**DOI:** 10.3390/ijms252313129

**Published:** 2024-12-06

**Authors:** Dominika Lewoń-Mrozek, Julia Kurzynoga, Piotr Jędrzejewski, Karolina Kędzierska, Alicja Partyka, Magdalena Kuriata-Kordek, Milena Ściskalska

**Affiliations:** 1 Department of Experimental Oncology, Hirszfeld Institute of Immunology and Experimental Therapy Polish Academy of Sciences, Rudolfa Weigla 12 St., 53-114 Wroclaw, Poland; dominika.lewon@hirszfeld.pl; 2 Student Society of Laboratory Diagnosticians, Wroclaw Medical University, Borowska 211A St., 50-556 Wroclaw, Poland; julia.kurzynoga@student.umw.edu.pl (J.K.); piotr.jedrzejewski@student.umw.edu.pl (P.J.); karolina.kedzierska@student.umw.edu.pl (K.K.); alicja.partyka@umw.edu.pl (A.P.); 3 Screening of Biological Activity Assays and Collection of Biological Material Laboratory, Wroclaw Medical University, 211A Borowska, 50-556 Wroclaw, Poland; 4 Department of Nephrology and Transplantation Medicine and Internal Diseases, Wroclaw Medical University, Borowska 213 St., 50-367 Wroclaw, Poland; magdalena.kuriata-kordek@umw.edu.pl; 5 Department of Pharmaceutical Biochemistry, Wroclaw Medical University, Borowska 211A St., 50-556 Wroclaw, Poland

**Keywords:** paraoxonase-1, oxidative stress, antioxidants, substrate specificity, genetic alterations, environmental factors, structure, single-nucleotide polymorphism, cardiovascular disease, lipids metabolism disorders

## Abstract

PON1 is a Ca^2+^-dependent enzyme that indicates a hydrolytic activity towards a broad spectrum of substrates. The mechanism of hydrolysis catalyzed by this enzyme is poorly understood. It was shown that the active site of PON1 is highly dynamic. The catalytic center of this enzyme consists of side chains of amino acids binding two calcium ions, from which the first one performs a structural function and the other one is responsible for the catalytic properties of PON1. This review summarizes available information on the structure of PONs, the role of amino acids located in the active site in specificity, and multiple substrate affinity of enzymes for understanding and explaining the basis of the physiological function of PONs. Moreover, in this paper, we described the changes in the structure of PONs induced by environmental and genetic factors and their association with diseases. The detoxification efficiency depends on the polymorphism of the *PON1* gene, especially Q192R. However, data on the association between single-nucleotide polymorphisms (SNPs) in the *PON1* gene and cardiovascular or neurodegenerative diseases are insufficient. The reviewed papers may confirm that PON1 is a very promising tool for diagnostics, but further studies are required.

## 1. Introduction

Human paraoxonases (PONs, EC number: 3.1.1.2) are Ca^2+^-dependent enzymes encoded by *PON1*, *PON2*, and *PON3* genes located in chromosome 7 (7q21.3-7q22.1) [1,2]. These genes are structurally very similar; about 70% similarity in nucleotide sequences and about 60% similarity in amino acid sequences have been demonstrated between these genes. The *PON1*, *PON2*, and *PON3* genes contain nine exons in their structure, but it is worth noting that PON1 has an additional codon in the fourth exon at position 106 (lysine) [3]. These genes encode the family of hydrolases that consists of three isoenzymes: paraoxonase-1 (PON1), paraoxonase-2 (PON2), and paraoxonase-3 (PON3) [1]. PON2 is considered to be the oldest evolutionary protein from the paraoxonase group [4]. Two isoforms of this protein have been observed: non-glycosylated PON2 (40 kDa) and glycosylated PON2 (43 kDa). However, no difference in their functions has been described so far [5]. PON2 is an intracellular enzyme, and its presence was demonstrated in many tissues, including the liver, kidney, lung, heart, and skeletal muscle [6]. In contrast to PON2, PON1 and PON3 are primarily synthesized in the liver and then secreted into the bloodstream, where they circulate in the blood in association with high-density lipoproteins (HDL) particles [1,2,7,8]. 

PON1 plays a key role in the formation and metabolism of HDL molecules. The complex of HDL-PON1 determines the antioxidant properties of HDL. Studies conducted by Aviram et al. [9] have shown that PON1 protects both HDL and LDL molecules from oxidation. The HDL-PON1 complex, as demonstrated in in vitro studies [10], is able to inhibit the oxidative reaction and reduce peroxides and aldehydes compared to HDL lacking the PON1 molecule. Therefore, the main protective mechanism linked to HDL-transported PON1 seems to be the reverse transport of cholesterol, preventing LDL oxidation [11]. The ability of PON1 to inhibit the lipid peroxidation process is possible due to the interaction of the free sulfhydryl group of the enzyme with oxidized lipids such as oxidized phospholipids, oxidized cholesterol ester, or lysophosphatidylcholine [9]. The presence of PON1 in the HDL structure contributes to the protection of blood vessel endothelial cells by an increase in the activity of endothelial nitric oxide synthase and the stimulation of nitric oxide synthesis [12]. It was demonstrated that HDL from PON1-deficient mice is not able to prevent LDL oxidation, whereas HDL from *PON1* transgenic animals protects LDL against oxidation more effectively than HDL from wild-type mice [13]. However, the biochemical basis for the putative antioxidative function of PON1 is still unclear [13,14,15]. 

This review summarizes available information on the structure of PON1, the role of amino acids located in active sites in specificity, and multiple substrate affinity of this enzyme for understanding and explaining the basis of the physiological function of PON1. Moreover, in this paper, we described the changes in the structure of PON1 induced by genetics and environmental factors and their association with the risk of disease based on lipid metabolism disorders.

## 2. Overall PON1 Structure in Relation to Its Substrate Specificity 

**Figure 1:**
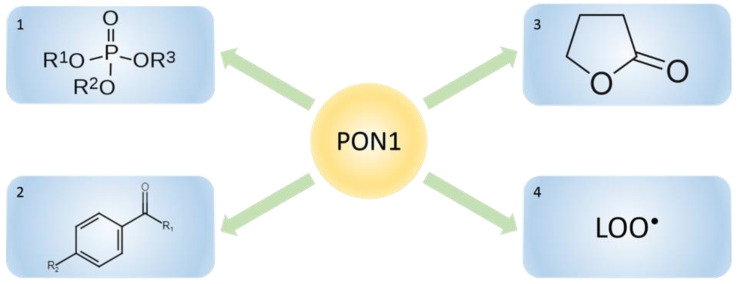
Substrates for PON1 activities: 1, organophosphates; 2, aryl esters; 3, lactones; 4, lipid peroxidates.

Human PON1 has three types of activity: phosphotriesterase, arylesterase, and lactonase activity [2,16]. Therefore, PON1 can hydrolyze (and inactivate) a variety of substrates, including aryl esters, thioesters, phosphotriesters, carbonates, lactones, and thiolactones (Figure 1) [2]. PON1 has three types of activity on several substrates, whereas PON2 and PON3 show high lactonase activity [11]. 

It was demonstrated that a native activity of PON1 is lactonase with γ- and δ-lactones as substrates [17]. It was shown that PON1-catalyzed lactone hydrolysis is not dependent on the pKa of the leaving group, unlike all other substrates. However, the rates of hydrolysis of aliphatic esters and aryl phosphotriesters are much slower and show a higher dependence on the pKa of the leaving group [17]. The lactonase activity of PON1 plays a significant role in the neutralization of a toxic metabolite in human blood–homocysteine thiolactone, formed when homocysteine is mistakenly selected by methionyl-tRNA synthetase in the process of protein biosynthesis [16,18]. The hydrolysis of homocysteine thiolactone catalyzed by PON1 prevents aggregation and the oxidation of LDL and the activation of macrophages [16]. In vitro studies [13] have shown that due to those features, PON1 is able to prevent atherothrombosis.

**Figure 2:**
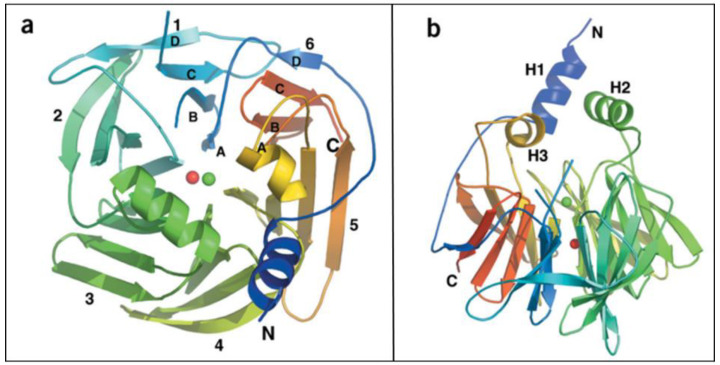
The structure of PON1. (**a**) View of the six-bladed β-propeller (each blade was marked as 1–6) A—inner β-strand of each blade, B, C— middle β-strands of each blade, D—outer β-strand of each blade, N—N-terminal residues of PON1, C—C-terminal residues of PON1. Ca^2+^ ions were marked in red and green color. (**b**) View of the propeller, including the three helices (H1–H3) involved in the binding of PON1 to cell membranes. Reprinted with permission from Ref. [20]. 2004, Springer Nature America, Inc.

**Figure 3:**
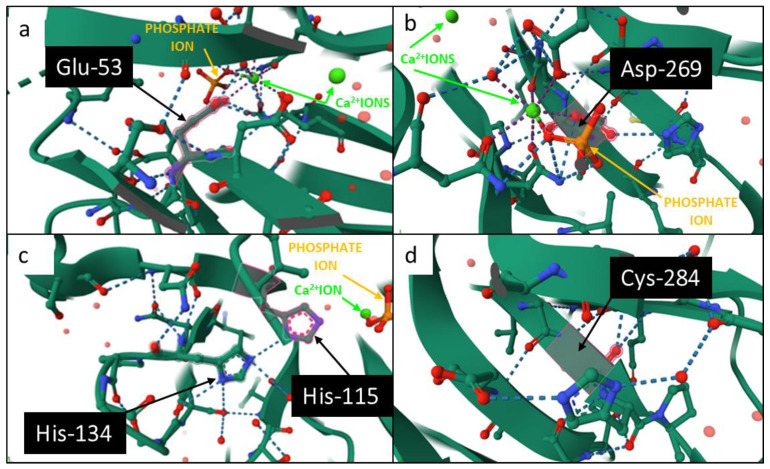
The structure of the PON1 active site takes into account amino acids essential for the catalytic base: glutamate (Glu-53) (**a**), aspartate (Asp-269) (**b**), histidines (His-134 and His-115) (**c**), cysteine (Cys-284) (**d**) (protein database (PDB) id. 1V04).

PON1 is a glycoprotein, and its final form consists of 355 amino acids. PON1 protein takes a conformation of a six-bladed β-propeller with a centrally located active site (Figure 2) [19,20]. The crystal structure of the PON1 active site is solved [2,21]. It is known that the enzyme catalytic center consists of two calcium ions chelated by side chains of glutamate at position 53 (Glu-53) and aspartate at position 269 (Asp-269), the phosphate ion, and a pair of catalytic histidines (located at position 115 and position 134 of the peptide chain) (Figure 3a–c) [20]. The calcium ion located near the surface of the active site is considered to play a catalytic role in fixing the orientations of the substrate and related residues (catalytic base). In addition, Asp- 269 is found to coordinate Ca^2+^ and facilitate the protonation of the alkoxide-leaving group by forming a hydrogen bond with a lactone [22]. The other Ca^2+^ is embedded at the bottom of the catalytic cavity, in the channel between blades, and it stabilizes the structure of the catalytic center [20]. The structural Ca^2+^ has a much higher affinity for its binding site and it is essential that it remains in the PON1 active site’s dimensional conformation. Dissociation of one of the calcium ions causes irreversible denaturation of the PON1 protein and thus the loss of its enzymatic activity [8].

On the other hand, divalent metal ions with higher binding affinities replace the native calcium ions at the PON1 active site. The in vitro studies showed an inhibitory effect of Cu^2+^, Zn^2+^, Mg^2+^, Mn^2+^, Ni^2+^, and Co^2+^ ions on PON1 activity [23,24,25,26]. It was reported that the partial inactivation of PON1 activity via Cu^2+^ ions may be caused by replacing the Ca^2+^ ion required for PON’s paraoxonase activity [23,27]. In another study, it was reported that Cu^2+^ ions totally inhibited the PON1 enzyme activity [25]. A decrease in PON1 activity caused by Zn^2+^ may be due to the absorption relationship between Ca^2+^ in the active site and Zn^2+^ cations [28] because the Ca^2+^ ions required for the stability and activity of the PON enzyme due to HDL in plasma are directly related to the absorption of Zn^2+^ ions [23,29]. Therefore, it is thought that these ions cause a decrease in activity by causing the inhibition of enzymatic activity [23]. However, it was determined that Mg^2+^ and Mn^2+^ ions did not show any effect on human PON1 enzyme activity [25]. In another study, it was reported that these metals may substitute Ca^2+^ without significantly altering the catalytic sites and contribute to forming a stable but inactive form of enzyme [30]. 

Metal substitution in PON1 can be also influenced by chelating agents, including EDTA. It was shown that the removal of Ca^2+^ ions from PON1 by the addition of EDTA results in the inactivation of Ca^2+^-dependent activities, including paraoxon and phenyl acetate hydrolysis; however, it does not cause loss of PON1 antioxidative capacity, including the ability of PON1 to protect LDL from oxidation [27,31,32,33]. This effect is probably mediated by the sequestration of calcium and the subsequent promotion of conformational changes that affect the enzyme activity [34]. It was shown that the effect of EDTA on PON1 activity can be restored by the addition of free calcium [24].

The molecular details of the enzyme action towards different substrates are also unclear [2,21]. It was shown that histidines at position 115 (His-115) and 134 (His-134) might play a role in the hydrolytic mechanism of PON1 towards lactones and arylesters. His-115 is presumed to function as a general base that disconnects hydrogen ions from the water molecule, thus generating the reactive hydroxide radical (OH•), which attacks the phosphoryl or carbonyl group of lactones and arylesters. His-134 is linked to His-115 via a hydrogen bond and increases the alkaline nature of His-115 and causes its activation. Another relevant element of the active site in terms of PON1 lactonase/arylesterase activity is the catalytic Ca^2+^. The Ca^2+^ function is able to stabilize an intermediate product of the OH• attack on the carbonyl group of the substrate [20,35]. The literature data on the importance of His-115 in PON1 catalytic activity are contradictory. It has been proposed that His-115 plays a pivotal role in carrying out the lactonase/arylesterase activities of the enzyme, and the replacement of histidine with valine at position 115 causes a significant decrease in these activities of the enzyme [36,37]. However, other studies [38] have shown that His-115 may not always be required for mediating the lactonase/arylesterase activity of the enzyme. Similarly, in the study conducted by Lin et al. [22], it was proven that His-115 can only form strong hydrogen bonding interaction with the attacking hydroxide to facilitate the hydrolysis (lactonase activity), but the catalytic base is provided by the glutamic acid at position 53 (Glu-53). Glu-53 can act without the assistance of His-115 to produce the hydroxide ion and activate the hydrolysis of substrates. Also, without the assistance of Glu-53, the activation of substrate hydrolysis by His-115 alone becomes more difficult [22]. Therefore, Glu-53 is necessary for hydrolysis, whereas His-115 is not essential but can promote the activity of PON1. It was shown that the replacement of Glu-53 with other amino acids results in the loss of the activity of PON1, and the mutation of His-115 causes the unaffected hydrolysis of dihydrocoumarin—a substrate for lactonase activity [22]. Additionally, it was demonstrated that the cysteine at position 284 (Cys-284), near the active site, might participate in the recognition and binding of the substrate (Figure 3d). This structure of the catalytic center ensures the stability of the PON1 enzyme [20].

**Figure 4:**
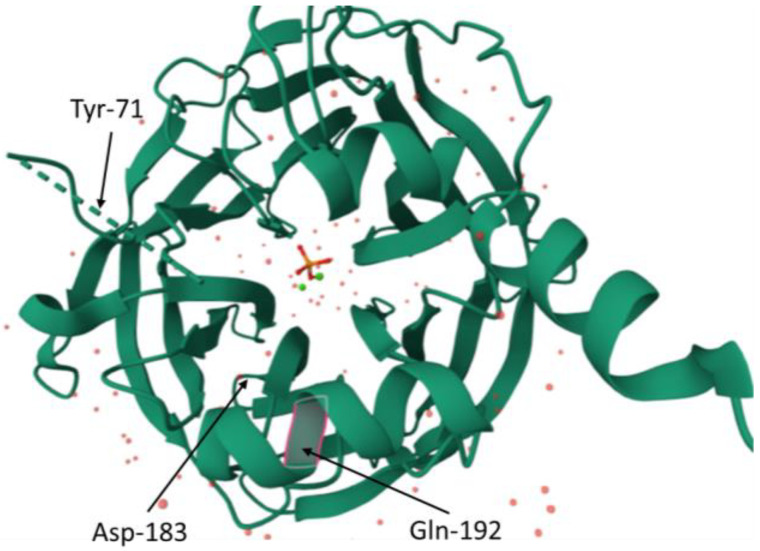
The amino acids engaged in hydrophobicity of the active site of PON1. Localizations of tyrosine at position 71 of the protein chain (Tyr–71), aspartic acid at position 183 of the protein chain (Asp–183) and glutamine at position 192 of the protein chain (Gln–192) were marked in red (protein database (PDB) id. 1V04).

It is known that amino acid residue, glutamine, at position 192 (Gln-192) plays an important role in the catalytic mechanism of enzymes. Mutagenesis studies indicate that different amino acid residues at position 192 engage different subsets in the active site of the enzyme by forming different hydrogen-bonding networks (Figure 4). This allows the substrates to adopt certain conformations (depending on the type of substrate) in the binding pocket of the enzyme by either inducing electrostatic or structural effects, thereby differentially affecting various enzymatic activities of PON1 [21]. This study suggests that, depending on the type of substrate, the presence of a particular amino acid residue at position 192 differentially alters the micro-environment of the active site of the enzyme, resulting in the engagement of different subsets of amino acid residues in the binding and the processing of substrates [21]. Therefore, the amino acid residue at position 192 is a determinant of the substrate specificity of the PON1 enzyme and its catalytic efficiency. The analysis of the mutations performed in the other study also indicates that the active site of PON1 is highly dynamic [2]. Moreover, the substitutions of amino acid residues far away from the active site can also modulate the hydrolytic activities of the enzyme [2]. 

**Figure 5:**
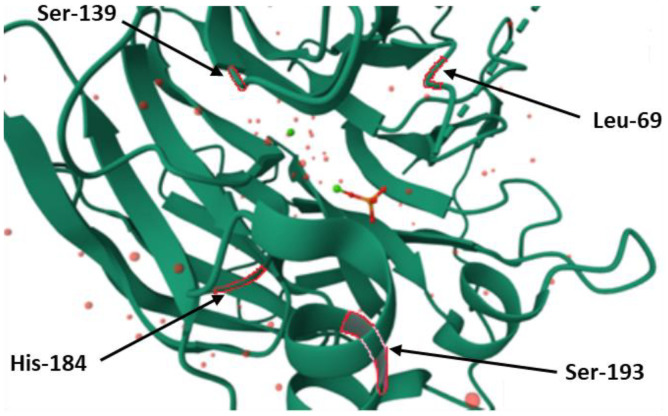
The amino acids providing the phosphotriesterase activity of PON1. Localizations of serine at position 139 of the protein chain (Ser–139), histidine at position 184 of the protein chain (His–184), leucine at position 69 of the protein chain (Leu–69) and serine at position 193 of the protein chain (Ser–193) were marked in red (protein database (PDB) id. 1V04).

PON1 was first known for its ability to hydrolyze phosphotriesters. The phosphotriesterase activity of the enzyme includes degrading agricultural organophosphorus pesticides such as parathion, paraoxon, diazinon, and chlorpyrifos [39,40,41]. In the study conducted by Cléry-Barraud [42], it was shown that arylesters and lactones are similarly positioned in the active site, whereas the organophosphates bind on another subunit. It was demonstrated that phosphotriesterase activity is influenced by the substitution of four amino acids, which are located near the active site. Replacing His-184, Leu-69, Ser-139, and Ser-193 caused a radical decrease in the hydrolytic activity of PON1 towards phosphotriesters, such as paraoxon (Figure 5). However, it is not clear which one of the mentioned amino acids plays the role of deprotonating the base, similar to His-115 in lactonase/arylesterase activity [35]. It is suggested that the hydrolysis of organophosphates, particularly in relation to paraxon, is a promiscuous activity of PON1, which is attributed to the considerable plasticity of the catalytic structure [39]. The aspect of the PON1 structure that is considered to have an impact on phosphotriesterase activity is a loop that includes amino acids in position 70–81. The loop is located above the active site and has variable conformation. Conformation changes result in different capacities of the active site and its availability for substrates and solvents. The amino acid residue that seems to play a vast role in stabilizing the conformation of the loop is tyrosine at position 71. The substitution of Tyr-71 results in decreasing hydrophobicity of the active site, which becomes more open. It increases the influx of water molecules inside the active site and disturbs its electrostatic balance. Tyr-71 also forms a hydrogen bond with Asp-183 above the active site, which enhances its hydrophobicity (Figure 4) [19].

### Structure of PON1 in Relation to Its Physiological Function

**Figure 6:**
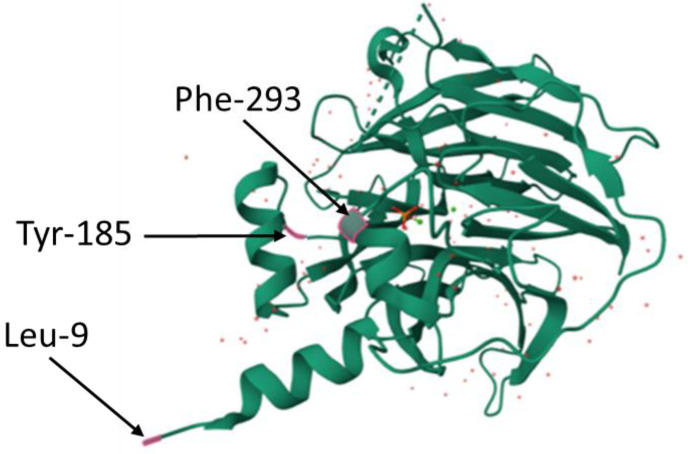
The most important amino acids for the binding of PON1 to HDL. Localizations of phenylalanine at position 293 of the protein chain (Phe–293), tyrosine at position 185 of the protein chain (Tyr–185) and leucine at position 9 of the protein chain (Leu–9) were marked in red (protein database (PDB) id. 1V04).

Studies investigating PON1 crystal structure revealed that PON1 is a six-bladed β-propeller with three hydrophobic helix parts on the surface of the protein molecule, which form a lid above the catalytic center. One of the helixes is actually a remaining N-terminal leader sequence, which is an interesting feature of PON1 [43]. This N-terminal leader sequence, together with two other helixes, anchors the PON1 molecule on the surface of an HDL particle or a cell membrane [44]. Helixes are hydrophobic due to tryptophan and tyrosine amino acids in their structure. It was shown that the most important amino acids for binding PON1 to HDL appear to be Leu-9, Tyr-185, and Phe-293 (Figure 6). Replacing those amino acids with glutamate or lysine resulted in a 1000-fold decreased affinity of PON1 to HDL [45]. PON1 interacts with HDL with the assistance of apolipoproteins apoA-I and apoE. ApoA-I and apoE are not required for PON1 binding to HDL, but it was shown that those apolipoproteins promote and stabilize PON1 activity [46,47]. Hydrophobic helixes on the surface of the PON1 molecule are also proposed to play a role in PON1 binding to cell membranes. PON1 interacts with cells of the immune system, especially monocytes and macrophages. It was shown that PON1 binds to cell membranes of macrophages in the same sites as HDL particles. Then, PON1 is internalized into the cytoplasm of a macrophage. Studies suggest that the ability of PON1 to bind to cell membranes via N-terminal sequence and phospholipids might have an impact on the antiatherogenic properties of the enzyme [48]. PON1 prevents the formation of foam cells by binding to macrophages and increasing the efflux of cholesterol from their cytoplasm [48]. This process occurs via the ATP-binding cassette transporter A1 (ABCA1) [49]. Moreover, PON1 can also contribute to a reduction in the amount of monocyte chemotactic protein (MPC-1) produced by endothelial cells in the course of atherosclerosis. Both purified PON1 and PON1 bound to HDL, due to their activity, prevent the formation of two compounds, 1-palmitoyl-2(5-oxovaleroyl)-sn-glycero-3-phosphoryl-choline (POVPC) and 1-palmitoyl-2-glutaroyl-sn-glycero-3-phosphorylcholine (PGPC), which stimulate the adhesion of monocytes to the endothelium and the production of MPC-1. This leads to the inhibition of MPC-1 synthesis and the weakening of monocyte chemotaxis [50]. It was also shown that PON1 can cause a decrease in secretion of cytokines, such as TNF-α and IL-6, by macrophages and inhibits differentiation of monocytes to macrophages [51,52].

Studies exploring interactions of PON1 with cell membranes revealed that PON1 may be carried into cell membranes by HDL and remains active on their surface [53]. Cells that acquire PON1 on their cell membrane appear to be less susceptible to oxidative stress and have an increased ability to neutralize 3-oxo-C12-homoserine lactone, which is a quorum-sensing molecule of bacteria, such as *Pseudomonas aeruginosa*. Therefore, PON1 activity is not completely dependent on HDL and remains, even if the enzyme is not attached to the lipoprotein particle [54]. 

PON1 is an enzyme that has antioxidative properties, although it is vulnerable to inactivation by oxidative stress. It is very well known that PON1 is involved in interactions with myeloperoxidase (MPO). Both enzymes form a ternary complex with HDL and influence each other during increased oxidative stress. PON1 inhibits oxidation by MPO, but on the contrary, MPO inactivates PON1. MPO catalyzes the synthesis of hypochlorous acid, which oxidates specific residues in the PON1 structure (Tyr-71) and apoA-I (Tyr-192). Oxidation of those residues impairs PON1 activity, stability, and ability of PON1 to bind to HDL [12,55].

## 3. Interaction Between PON1 and Heavy Metals 

Heavy metals are common environmental pollutants and have been proven to negatively influence human health. Heavy metal poisoning may be a result of occupational hazards, industrial pollution, food and water, and smoking. The main mechanism of heavy metal toxicity is the disruption of the oxidative balance in the organism by excessive free radical formation, which leads to oxidative stress [56]. PON1 plays a role in oxidative stress elimination. Its activity and concentration seem to be influenced by heavy metals. One of the main elements that inhibit PON1 activity (purified as well as HDL bound) is copper. Lactonase activity is the most vulnerable to inactivation by copper ions. It may be restored by catalase or dietary lipids, such as oleic acid and phosphatidylcholine. Restoration of PON1 lactonase activity by native catalase can confirm that the putative mechanism that stands behind the inactivation of PON1 lactonase activity is an oxidative modification of lactonase activity center by copper ion-bound hydroxyl radicals [57,58]. 

PON1 may play a role in heavy metal detoxification. PON1 was found to increase its concentration in rat livers after treatment with a lead, cadmium, and mercury mixture [59]. Data suggest that a slightly elevated concentration of lead increases PON1 activity, whereas major exposure to this metal decreases the activity of the enzyme in a dose-dependent manner [60,61]. Other studies also revealed that common environmental exposure to lead and cadmium does not impair PON1 activity, although an excessive dose, acquired via, e.g., cigarette smoking, is significantly associated with lower hydrolytic activity of the enzyme [62]. Other studies also show that elevated blood concentrations of metals such as lead, cadmium, and mercury significantly decrease PON1 activity and suggest a putative mechanism of PON1 inhibition. It may be the consequence of divalent metal ions’ ability to bind to PON1 molecules via free sulfhydryl groups located on amino acid residue Cys-285 [60]. Another metal that decreases PON1 activity is zinc. The putative mechanism that stands behind PON1 inhibition by zinc ions is the binding this metal to histidine residues, including His-115 and His-134, which are essential amino acids in PON1 active sites [60]. 

PON1 activity is decreased by mercury or its metal–organic compound, methylmercury, which was revealed in studies investigating the influence of metals on PON1 activity in Inuit, who are exposed to mercury and methylmercury due to their diet that is rich in marine mammals and other sea foods. However, a traditional Inuit diet is also rich in selenium. The concentration of selenium is positively correlated with PON1 activity, which might suggest that this element acts on the contrary to mercury and abolishes its toxic effects [63,64]. Selenium is a cofactor in many antioxidant enzymes and may play a role in reducing total oxidative status and protecting other protein molecules, such as PON1 [65]. A positive correlation between selenium and PON1 levels was also found in obese children. Obesity is a condition with an increased risk of excessive oxidative stress, which is harmful to PON1 activity. Data suggest that selenium plays a role in maintaining the oxidative balance of systems, which include PON1 [66]. Mercury may also influence PON1 activity via epigenetics. It was demonstrated that prenatal exposure to mercury leads to the hipermethylation of the *PON1* gene in cord blood, which causes poorer cognitive test scores [67]. 

The interaction between PON1 and iron remains vague. In vitro studies of purified PON1 revealed that iron ions significantly inhibit PON1 activity [68]. PON1 activity is lower in patients with iron deficiency and increases after iron treatment [69]. However, in patients with normal iron homeostasis, iron supplementation results in a decrease in PON1 activity [70]. Those data come from studies conducted in groups of adult patients. In children with iron deficiency, there were no interactions between iron level and PON1 activity observed [71]. Patients suffering from hemochromatosis, which manifests itself with iron overload, have significantly lower PON1 activity, and its decrease is inversely correlated with ferritin level. Moreover, there is a higher expression of PON1 in the liver of hemochromatosis patients, especially in the most inflamed areas of the liver tissue. Those data indicate that PON1 plays an important role in iron detoxification, and its activity is affected by iron level abnormalities [72]. 

## 4. Genetic Alterations of PON1 Structure

**Figure 7:**
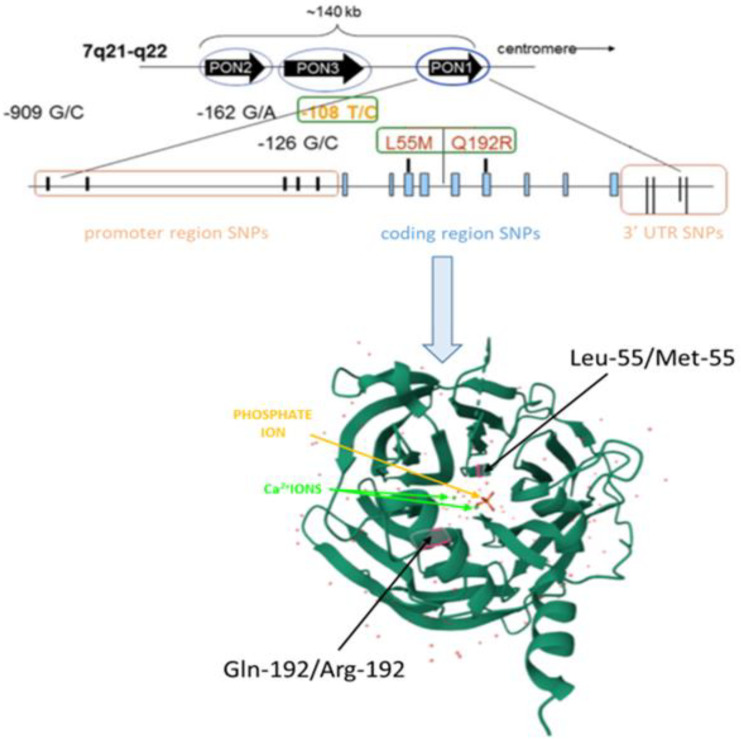
Localization of the Q192R and L55M polymorphisms in the *PON1* gene and their implication on the changes in enzymatic structure in the PON1 molecule. The replacement of glutamine with arginine at position 192 of the protein chain (Gln-192/Arg-192) and the replacement of leucine with methionine at the protein chain position 55 (Leu-55/Met-55) were marked with red lines. Adapted with permission from Ref. [8], 2015, Elsevier B.V. and the protein database (PDB, id. 1V04).

PON1 catalytic efficiency for hydrolysis organophosphates and lactones is modulated by the Q192R (rs662) polymorphism [40,73,74,75]. The Q192R polymorphism is obtained by the mutation of thymine to cytosine in codon 192, which is followed by the replacement of glutamine with arginine at position 192 of the protein chain (Figure 7) [8]. The PON1 Q192 allele is less efficient in hydrolyzing organophosphates than the PON1 R192 allele [40]. In addition, it was shown that maternal PON1 status (PON1 activity and functional Q192R phenotype) modulates the detrimental effects of exposure to the organophosphates on fetal gene expression and biomarkers of exposure [40]. The difference in PON1 activities in relation to phenotypes for Q192R polymorphism was also shown in the blood of healthy people environmentally exposed to cadmium, lead, and mercury. Studies show that PON1 activity in subjects with the RR genotype is more susceptible to toxicity of heavy metals, including lead and cadmium [76]. Moreover, those differences seem to be manifested in a dose-dependent manner when intensified, e.g., occupational, exposure is considered [61]. 

A PON1 polymorphism, L55M (rs854560), is a mutation of adenine to thymine and is manifested by the replacement of leucine with methionine at the protein chain position 55 (Figure 7) [8,21,77]. It has not been shown yet that the detoxifying activities of PON1 are affected by L55M, although L55M, as well as Q192R, affects PON1 activity towards homocysteine thiolactone. The L55M polymorphism was found to have a much smaller effect on PON1 activity than the Q192R polymorphism. The differences between them concern the affinity and catalytic activity of substrates. It has also been reported that the occurrence of the PON1 Q192R polymorphism determines the lower effectiveness of PON1 in inhibiting LDL oxidation, which is associated with a weakened ability of the enzyme to hydrolyze lipid peroxides. Studies conducted by Aviram et al. [78] have shown that PON1 192Q has higher hydrolytic and antioxidant efficacy compared to PON1 192R. It was shown that the 192R allele is associated with a higher level of oxidized LDL and a lower ability to neutralize oxidized lipids in atherosclerotic lesions; therefore, it is less efficient at protecting against cardiovascular disease [79,80]. It has also been claimed that PON1 polymorphisms (C-108T, M55L, G-162A, R-160G,) are associated with coronary artery disease [81,82,83,84]. However, in other studies, it was pointed out that the PON1-Q192R genotype, although strongly influencing PON1 activity, is not associated with the risk of CAD [85,86]. Moreover, it has been pointed out that there is a possible association between the 55L and 192R alleles and the severity of CAD [86,87]. 

In other studies, it has been shown that 55L and 192R variants hydrolyze homocysteine thiolactone more efficiently than 55M and 192Q variants [88]. Other studies focused on the relationship between oxidative stress and the PON1 genotype, and PON1 activity showed that individuals with the RR192 genotype had lower levels of systemic oxidative stress markers than those with the QQ192 genotype. It has been reported that alloenzyme Q192 binds to the HDL particle with a 3-fold lower affinity than alloenzyme R192. This contributes to its lower stability and reduced lipolactonase activity. It has also been claimed that in individuals with the arginine (R) mutation at position 192, the incidence of coronary heart disease and the risk of serious cardiovascular events are reduced [89].

In other studies, the association between the C-108T and G-909C promoter region polymorphism and the presence of coronary heart disease (CHD) was analyzed. It was observed that PON1 activity and concentration were significantly lower in the group of CHD patients compared to the control group, regardless of their C-108T and G-909C genotype [90]. It was shown that the association between CHD and low serum PON1 activity was an independent factor of new CHD events [91]. 

Cardiovascular disease and abnormalities in lipid metabolism are predictive factors for Alzheimer’s disease [92], initiated by the deposition of beta-amyloid plaques on the outside of neurons and tau protein tangles inside neurons in the brain [93]. The occurrence of PON1 in plasma and cerebrospinal fluid (CSF), as well as its antioxidant function, focused the interests of researchers on verifying a potential link between PON1 polymorphisms and the risk of developing neurodegenerative diseases [94]. The association between the A allele of the rs705379 PON1 polymorphism with Alzheimer’s disease in a Caucasian population was observed [95]. Simultaneously, it was also shown that the GG genotype of this polymorphism was associated with a reduced risk of AD in Caucasians [95]. Moreover, in these studies, no significant association between the L55M, Q192R, and 161C/T polymorphisms in the *PON1* gene and AD was found [95]. Pi et al. also indicated no association between the L55M and Q192R PON1 polymorphisms and AD [96]. In 2017, Saeidi et al. showed that the T allele was the most common allele for people with AD for the -108C> T PON1 polymorphism. However, the highest PON1 enzymatic activity was found for the CC genotype [97].

In 2019, Verde et al. showed that the Q192R PON1 polymorphism may exhibit a gene effect modifying the course of amyotrophic lateral sclerosis (ALS) [98]. In contrast to previous single studies, Wills et al. in their meta-analysis show no significant association of PON1 polymorphisms with amyotrophic lateral sclerosis [99]. Lee et al. also showed no association between the polymorphisms in the *PON1* gene (Q192R and L55M) and the occurrence of ALS in the European population [100].

## 5. Materials and Methods

A comprehensive review was performed by systematically analyzing the literature published up to December 2023. The PubMed (https://www.ncbi.nlm.nih.gov/pubmed/, accessed on 31 December 2023) and Google Scholar (https://scholar.google.com/, accessed on 31 December 2023) databases were searched for variations of keywords as follows: ‘paraoxonase-1’, ‘structure’, ‘activity’, ‘antioxidants’, ‘oxidative stress’, ‘substrate specificity’, ‘physiological function’, ‘environmental factors’, ‘genetic alterations’, ‘single nucleotide polymorphism, ‘cardiovascular disease’, and ‘lipids metabolism disorders’. We also used PubMed Advanced Search Builder to search for the following phrases: (paraoxonase-1) AND (genetic alterations) AND (lipids metabolism disorders); (paraoxonase-1) AND (structure) AND (activity) OR (substrate specificity) and others.

The literature search was performed by five authors. We found more than 5000 records that were screened by the authors. We included 89 papers, which specifically focused on the paraoxonase-1 and lipids metabolism disorders. Abstracts in a language other than English were excluded. Editorial comments, letters to the editor, or indexed abstracts at international congresses were not considered. The paper based on cellular, animal, and human studies was included. We included the meta-analyses, and review articles were included. Formal institutional review board (IRB) approval for this study was not required.

## 6. Conclusions and Future Directions

Our work summarizes the findings focused on the structure of PON1 in relation to its physiological function. It is known that essential amino acids in the structure of PON1 include His-115, Glu-53, Cys-284, and Gln-192. Cys-284 is responsible for the recognition and binding of the substrate, which is accompanied by catalytic Ca^2+^ ions. His-115 and Glu-53 are directly involved in hydrolytic reactions. Gln-192 plays an important role in docking particular substrates in the active center. The substrate specificity of PON1 is influenced by the plasticity of the loop above the active center determined by Tyr-71, which provides the electrostatic balance. PON1 is bound to HDL via the N-terminal leader sequence and two other hydrophobic helixes and provides antioxidative properties of HDL. PON1 plays an important role in the detoxification of organophosphorus insecticides and probably heavy metals. The detoxification efficiency depends on the polymorphism of the *PON1* gene, especially Q192R. However, data on the association between SNPs in the *PON1* gene and cardiovascular or neurodegenerative diseases are insufficient and contradictory. There is a need for further studies in this field to clearly determine the influence of SNPs in the *PON1* gene on the risk of diseases associated with lipid metabolism disorders. The reviewed papers may confirm that PON-1 is a very promising tool for diagnostics, but further studies are required. 

## Data Availability

No new data were created or analyzed in this study. Data sharing is not applicable to this article.
